# Is It Time to Reconsider the U.S. Recommendations for Dietary Protein and Amino Acid Intake?

**DOI:** 10.3390/nu15040838

**Published:** 2023-02-06

**Authors:** Mary Weiler, Steven R. Hertzler, Svyatoslav Dvoretskiy

**Affiliations:** 1 Scientific and Medical Affairs, Abbott Nutrition, 2900 Easton Square Place, Columbus, OH 43219, USA; 2 Department of Kinesiology and Community Health, University of Illinois, Urbana-Champaign, IL 61801, USA

**Keywords:** protein, RDA, nitrogen balance, indicator amino acid oxidation, amino acids

## Abstract

Since the U.S. Institute of Medicine’s recommendations on protein and amino acid intake in 2005, new information supports the need to re-evaluate these recommendations. New lines of evidence include: (1) re-analysis/re-interpretation of nitrogen balance data; (2) results from indicator amino acid oxidation studies; (3) studies of positive functional outcomes associated with protein intakes higher than recommended; (4) dietary guidance and protein recommendations from some professional nutrition societies; and (5) recognition that the synthesis of certain dispensable amino acids may be insufficient to meet physiological requirements more often than previously understood. The empirical estimates, theoretical calculations and clinical functional outcomes converge on a similar theme, that recommendations for intake of protein and some amino acids may be too low in several populations, including for older adults (≥65 years), pregnant and lactating women, and healthy children older than 3 years. Additional influential factors that should be considered are protein quality that meets operational sufficiency (adequate intake to support healthy functional outcomes), interactions between protein and energy intake, and functional roles of amino acids which could impact the pool of available amino acids for use in protein synthesis. Going forward, the definition of “adequacy” as it pertains to protein and amino acid intake recommendations must take into consideration these critical factors.

## 1. Introduction

Recommended total dietary protein intakes for adults have remained constant for over 40 years and individual amino acid intake recommendations have not been updated since reports published in the 2005–2007 timeframe [1,2,3,4]. Nitrogen balance determinations comprise much of the scientific evidence for establishing protein requirements. However, there are multiple difficulties associated with the nitrogen balance methodology regarding not only the technical aspects of how to measure it, but also the interpretation of the data. Whole-body protein turnover, as evidenced by enzyme kinetics of the urea and citric acid cycles, strongly suggests that the components of protein turnover, including amino acid oxidation, are affected and may even be regulated by changes in amino acid supply and/or amino acid concentration [5]. Evidence of alternative roles of amino acids beyond their role as subunits of protein synthesis indicate that a competitive scenario for the available amino acid pool could likely impact the homeostasis measured in a nitrogen balance study [6]. In addition, metabolic adaptation to low protein intakes complicates the interpretation of nitrogen balance data [7]. Finally, studies on histidine demand have shown that nitrogen balance is not equivalent to amino acid balance [8]. These considerations, along with the understanding that the nitrogen balance technique has many limitations including overestimation of nitrogen intake and an underestimation of nitrogen excretion [9], have led to alternative approaches to understanding protein needs.

In the latest Dietary Reference Intakes (DRI) [3] and WHO/FAO report [4] on protein and amino acid intake recommendations, it was acknowledged that the 24-h indicator amino acid oxidation (IAAO) and balance method was considered the gold-standard method to estimate amino acid requirements [3,4]. New studies using this methodology have supported increased needs for both total protein and for some amino acids for some populations. With the understanding that nitrogen balance is not equivalent to amino acid balance, this review also explored whether information on usual recommended intakes of protein versus those exceeding the Recommended Dietary Allowance (RDA) support improved health or functional outcomes, as this is the public health aim for setting a nutrition requirement, to determine if these lines of alternative evidence further validate the current U.S. DRI for protein or lend credence to increasing the RDA [3].

Three populations warrant this scrutiny as their protein needs are impacted by special metabolic demands: older adults, young children, and the pregnant and lactating populations. In addition, limited IAAO data suggest that both strength and endurance athletes require considerably more protein than the RDA [10,11,12,13], even though the current RDA makes no adjustments for physical activity compared with non-exercising adults. Evidence supporting the need for re-evaluation of protein requirements includes: (1) revised interpretations of the nitrogen balance data upon which protein intake recommendations have been based; (2) new indicator amino acid oxidation studies in several populations; (3) investigations of the health and functional benefits of higher-than-typically recommended protein intakes in older adults; (4) how both essential and non-essential amino acids should inform amino acid recommendations; and (5) alternative perspectives for expressing protein and/or amino acid recommendations (e.g., percent of dietary energy, per meal protein requirements). The lines of evidence have prompted some prominent researchers and scientific societies to recommend, for some healthy populations (e.g., athletes, older adults), protein intakes that are substantially higher than established recommendations from the U.S. Institute of Medicine/National Academy of Medicine or the Food and Agricultural Organization of the United Nations [14,15]. Extending higher recommendations should also be considered for other healthy populations, women who are pregnant or breastfeeding, where this question was addressed at a recent National Academies of Sciences workshop on nutrition during pregnancy and lactation [16]. Reconsideration of higher protein recommendations should also be applied to children ≥ 3 years of age, or at minimum a caveat statement incorporated that addresses the level of protein quality, with special consideration for those at risk for malnutrition or undernourished [17]. This paper will review each of these lines of evidence and provide a rationale for the need to re-evaluate the formal recommendations for protein and amino acid intake.

## 2. Pros and Cons of Nitrogen Balance Determinations

The current U.S. adult Recommended Dietary Allowance (RDA) for protein (0.8 g/kg body weight/d) has changed very little since the RDA concept was first developed in 1941, hovering around 1.0 g/kg body weight/d at that point [1,2,3]. Much of the rationale underpinning the current protein RDA has focused on the maintenance of whole-body nitrogen balance. Nitrogen balance reflects the difference between nitrogen intake and the amount excreted in urine, feces, and miscellaneous sources such as sweat, hair, nails, and secretions [18]. For many years, nitrogen balance studies in humans have been the gold standard for determining protein requirements. Despite its long history of use in the determination of protein requirements, the nitrogen balance technique is associated with multiple significant limitations, including [18]: (1) errors toward positive nitrogen balance due to overestimation of dietary nitrogen intake and underestimation of nitrogen losses from the body; (2) long periods of adaptation (e.g., 5–7 days) that are required to equilibrate the body’s nitrogen pool when protein intake is altered; (3) influence of dietary energy/carbohydrate intake that can alter utilization of amino acids for energy; and (4) use of simple linear regression analysis for data interpretation when, in reality, the relationship between nitrogen intake and body nitrogen losses is not linear (the efficiency of protein utilization decreases as zero nitrogen balance is neared) [19].

Rand et al. [20] conducted a meta-analysis of 58 nitrogen balance studies in adults for the purpose of estimating the protein requirement of healthy adults. These studies were classified as Estimation, Test, and Obligatory studies, based on differences in protocol design. The Estimation studies evaluated multiple nitrogen intakes (at least 3) near purported requirements, each test intake was fed for 10–14 d, and fecal and urinary nitrogen data from the last 5 days were used to represent the response to that intake. The Estimation studies were subdivided into: (1) 19 primary studies, in which multiple nitrogen intakes were evaluated within a given individual and data were presented per individual subject; and (2) 8 secondary estimation studies in which multiple nitrogen intakes were evaluated but only grouped data were presented or data from different persons at different intakes were reported. The Test (*n* = 17) studies evaluated 1 or 2 nitrogen intakes and were not specifically designed to estimate overall protein requirements and the Obligatory (*n* = 14) studies measured obligatory or endogenous nitrogen losses on very low protein diets. Because of the variability in study designs and purposes, the analysis for determining the RDA was restricted to the 19 primary estimation studies. Because a graphical representation of the nitrogen intake data for these 19 studies showed no evidence of nonlinearity, a simple linear regression model was employed to identify the point of zero balance. Use of this approach resulted in an estimated average requirement (EAR) and RDA of 0.66 and 0.83 g protein/kg body weight/d, respectively, for adults. These values (RDA rounded down to 0.8 g/kg body weight/d) were utilized by the U.S. Institute of Medicine (now renamed as the National Academy of Medicine) to derive the Dietary Reference Intakes (DRI) for protein in 2005 [3]. Based on this meta-analysis, the World Health Organization (WHO) also adopted the adult protein intake recommendation of 0.8 g/kg body weight/d in 2007 [4].

The use of single linear regression analysis to interpret the nitrogen balance data, used in the development of the protein RDA, is interesting for several reasons. First, the single linear regression model ignores that nitrogen retention by the body cannot continually increase as nitrogen intake increases. At some point, there will have to be a period of zero-slope, or the body would simply continue to gain nitrogen in perpetuity as protein intake increases, which is not the case. Second, research on lysine requirements has demonstrated that the relationship between lysine intake and nitrogen balance is curvilinear and that statistical models are needed to account for this nonlinearity [19,21]. These could include square root transformation of nitrogen intake data in a linear regression model or exponential asymptotic regression. However, Rand et al. [20] defended the use of single linear regression on the basis that there was no evidence of nonlinearity in primary determination studies gathering nitrogen balance data at intakes relatively close to those that might be expected to produce zero balance (Figure 2 of reference [20]). The concern with this contention is the authors’ selection of a range protein intakes that might be expected to produce zero balance was perhaps too narrow. For nitrogen intakes up to around 200 mg nitrogen/kg body weight per day, which corresponds to a protein intake of around 1.25 g/kg per day, the relationship between nitrogen intake and nitrogen balance appears to be linear. This approach, though, neglects the effects of multiple data points at nitrogen intakes around 250 mg/kg/d on the model (intakes that could be present in some segments of the healthy population, such as highly active individuals). When Rand et al. [20] included data from all study types (obligatory, test, and estimation), the nitrogen intake data appeared to look more curvilinear (Figure 4 of reference [20]). The curvilinear appearance of the data distribution and the appropriateness of applying different statistical approaches to the data from the combined data (58 studies), including mathematical transformations and biphasic linear regression, was explored. However, it was judged that only the data from the primary estimation studies would be used to derive the requirement estimate, with the justification that the protocols from the different types of studies were not properly comparable and derived lines of different slopes in the statistical analysis.

Humayun et al. [22] reanalyzed the nitrogen balance data from these same 19 primary estimation studies of Rand et al. [20], combined with data from 7 of the secondary estimation studies. Humayun et al. [22] also included 3 studies not covered in the Rand et al. meta-analysis. Humayun et al. then applied the two-phase linear regression model and reported a mean daily nitrogen requirement of 146 mg nitrogen/kg (0.91 mg protein/kg), with an upper 95% confidence interval bound of 158 mg nitrogen/kg (0.99 g protein/kg). Thus, the use of the two-phase regression analysis results in protein requirement estimates that are 38% higher than when simple linear regression is applied.

## 3. Results from Indicator Amino Acid Oxidation Studies for Determining Protein Requirements

**Figure 1:**
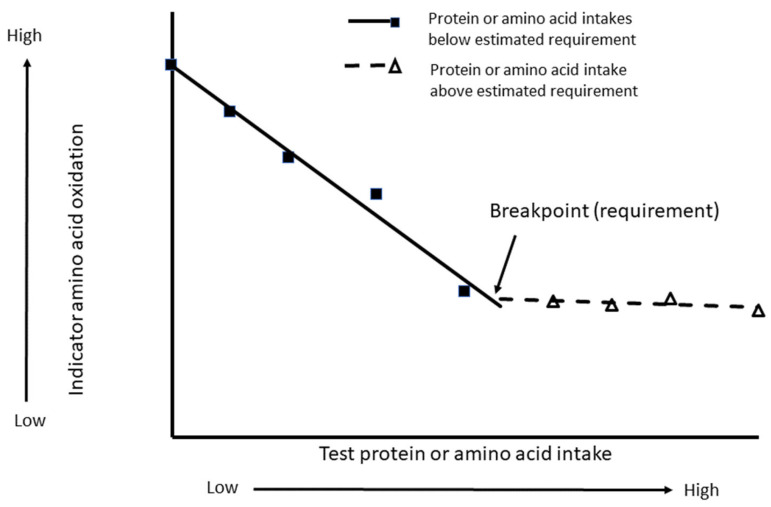
Determination of a test amino acid requirement based on the oxidation of an indicator amino acid (theoretical data) and employing the biphasic linear regression technique.

Given the technical difficulties of performing nitrogen balance studies, alternative methods of estimating amino acid or protein requirements have been employed. One of these is the indicator amino acid oxidation (IAAO) method. This method was developed in the 1980s by Ball and Bayley [23,24,25,26] to determine amino acid requirements in growing piglets and was later adapted for measurement of amino acid requirements in humans [27]. The principle of this method is depicted in Figure 1 and has been previously reviewed [28,29]. In essence, a diet deficient in a particular essential amino acid (aka the limiting amino acid) restricts protein synthesis. As such, the supply of essential amino acids other than the limiting amino acid exceeds their incorporation into protein. Given that amino acids are not stored in the body, oxidation of the other essential amino acids increases. In the IAAO method, a stable carbon isotope of an “indicator” amino acid, typically phenylalanine, can be administered to human subjects along with diets that vary in amounts of protein, or the amino acid of interest and the excretion of labeled carbon dioxide can be monitored in the breath as a measure of oxidation. As the intake of the test amino acid approaches the requirement, oxidation of the indicator amino acid steadily decreases until it reaches a nadir. Further increases in the test amino acid intake result in no further decreases in the oxidation of the indicator amino acid. As with the nitrogen balance technique, biphasic linear regression can be applied, resulting in one line with significant negative slope (test amino acid intakes below the requirement) and one line with an essentially zero slope (test amino acid intakes above the requirement). The point at which these two lines converge is termed the “break point” and represents the amino acid requirement.

The IAAO method has also been utilized to estimate daily protein requirements. Humayun et al. [22] reevaluated the protein requirement of young men in two ways: (1) researchers reanalyzed the existing nitrogen balance data upon which the present RDA is based [20] but using biphasic linear regression instead of simple linear regression; and (2) conducted an IAAO study in 8 healthy young men receiving graded protein intakes, with phenylalanine as the indicator amino acid. Interestingly, the reanalysis of nitrogen balance data resulted in a breakpoint/requirement of 0.91 g protein/kg/d, with a population safe intake (upper 95% confidence interval (CI), interpreted similarly to the RDA) of 0.99 g protein/kg/day. The IAAO study resulted in a similar breakpoint/requirement of 0.93 g protein/kg/d, with a population safe upper 95% CI of 1.24 g protein/kg/d. Thus, the RDAs derived from the re-analysis of nitrogen balance data and the IAAO method were 16 and 55% higher, respectively, than the present protein RDA. Since Humayun et al. [22] the protein requirements of several different populations have been evaluated using the IAAO methodology (See Table 1). In each of these studies, the IAAO method indicates a considerably higher population safe protein requirement than the current RDA, except for 2 studies of young Chinese adults where the IAAO resulted in a lower RDA than the current Chinese recommendation, yet higher than the U.S. RDA protein requirement. Note from Table 1 that although the current protein RDA makes no additional allowances for physical activity, the IAAO method results in recommendations that are at least double and nearly triple the RDA for both strength and endurance athletes.

## 4. Historical Perspectives on Higher Protein Recommendations Align with IAAO Estimates

There have been discussions of the strengths and limitations of the IAAO method vs. other methods for determining protein and amino acid requirements [9,40], but, on balance, the IAAO method, either short-term or extended to a 24-h balance, is probably the preferred approach [41]. Even so, some may question whether protein recommendations so much higher than the present RDA are warranted and, if so, in which populations. One approach is to seek some degree of corroborative observational evidence by reviewing the functional and health benefits of protein intakes higher than the RDA. Scrimshaw authored reviews in 1976 providing some history behind the derivation of protein recommendations [42] (pp. 136–142; pp. 198–203), In these reviews, Scrimshaw noted that in 1881 the German scientist Voit, based on experimentation and observation, recommended that a 70-kg man consume 118 g protein and 3000 kcal per day. This was closely aligned to an earlier recommendation by Playfair (1865) of 119 g/d and the 1894 recommendation of 125 g/d by Atwater. By 1935, the Technical Committee on Nutrition of the League of Nations concluded that the protein intake for all adults should not fall below 1 g protein per kg body weight per day, advocating protein intake from a variety of sources with part coming from animal foods. It is interesting that these estimates that arose before the widespread use of nitrogen balance studies agree well with results of recent IAAO studies. Similarly, a cross-section of Dutch athletes reported mean protein intakes of 90–108 g/d (1.4 to 1.5 g/kg/d) in males and females, respectively, that are well above the RDA [43]. Protein recommendations have come down to the present RDA in the era of nitrogen balance testing, but it is not clear that such changes have been optimal for health and physical function.

## 5. Support for Higher Protein Recommendations for Older Adults (≥65 Years) Based on Evaluation of Studies Published in the Last 20 Years which Measure Health and Functional Outcomes

Typically studies on protein needs and aging recruit cohorts 65 years or older; however, concerns for muscle quality have motivated some researchers to include “younger-old” individuals e.g., 40 years plus. The protein needs of older individuals and the suitability of the RDA has come under question in recent years because, in this population: (1) the prevalence of sarcopenia varies from 9.9 to 40.4% [44]; and (2) there appears to be considerable anabolic resistance associated with aging, requiring a greater amount of protein to stimulate a given rate of muscle protein synthesis compared with younger adults [45]. Age-related anabolic resistance to amino acids and protein amounts to reductions of nearly 40% efficiency in muscle protein synthesis. The two general, nonexclusive approaches for treating or preventing sarcopenia include increase in intake of amino acids and/or protein by older individuals and treatments to increase the anabolic sensitivity of older skeletal muscle to amino acids [46]. Although the RDA is not specifically adjusted for the effects of aging, numerous studies, both observational and interventional, have shown benefits of higher protein intakes in older people for preserving strength, improving body composition, and general health and physical function (See Table 2).

These functional and body composition data, mainly from older adults, tend to support the estimates derived from IAAO studies suggesting that the protein RDA is too low for optimal function in this population. Hudson et al. [65] conducted a meta-analysis of 18 studies comparing protein intakes equivalent to the RDA versus above the RDA. This meta-analysis included 13 studies in which either a catabolic stressor (i.e., energy restriction) or anabolic stressor (i.e., resistance training) was present, and 5 studies in which no stressor was present. When the data were considered as a whole, the authors reported that protein intakes above the RDA benefitted changes in lean body mass relative to consuming the RDA for protein. However, when the authors separated out the 5 studies (7 treatment groups) [66,67,68,69,70] having neither an anabolic nor catabolic stressor, they reported that the protein RDA was adequate for supporting lean mass in these individuals. There are several interesting points to make regarding the interpretation of these data. First, many older adults experience some degree of energy restriction due to multiple factors (e.g., illness, social isolation, poor appetite), which could be an argument supporting a higher protein RDA in this population. Second, among the 5 studies with no anabolic or catabolic stressors, other important health indicators may have been overlooked. For example, one of these studies [66] showed significant benefits of higher protein intake (2 X RDA vs. RDA) on lean body mass and leg power in elderly men. In each of the other 4 studies showing the protein RDA to be adequate for support of lean body mass, other ancillary benefits of protein intakes above the RDA were reported, including lower body fat mass/better weight maintenance following weight loss [67,68,69], reductions in blood lipids (triacylglycerols, total- and LDL-cholesterol) when the additional protein source was whey [70], and improved appetite control, again when whey protein was the additional protein source [69]. Finally, in 3 of these studies, the mean age was under 50 years, so these ancillary benefits of higher protein intake may not be restricted to older adults.

## 6. “Per Meal” Protein Requirements

Although virtually every protein recommendation has been based on achieving a specific daily protein intake on a body weight basis, some authors have suggested the use of “per meal” protein requirements [71,72]. This approach is largely based on the maximal stimulation of postprandial muscle protein synthesis (MPS) in healthy adults for a given meal protein intake. The amount of meal protein intake that maximizes postprandial MPS varies, depending on factors such as age of the individual, amino acid content of the protein (especially leucine as a stimulator of muscle protein synthesis), and presence or absence of resistance training exercise [71,73,74,75]. For example, maximal postprandial MPS ranges from about 0.24 to 0.4 g/kg body weight (19–32 g for an 80-kg individual) [45], with the lower end of the range reflecting requirements for young people and the higher end of the range applying to older individuals, who often experience considerable anabolic resistance. It has been proposed that failing to meet optimal per meal protein intakes can impair postprandial and potentially daylong MPS. For example, Mamerow et al. [76] evaluated, in a young adult population, isonitrogenous diets in which the protein intake was evenly divided over the course of 3 meals (about 30 g protein per meal) vs. a “skewed” distribution pattern (11 g, 16 g, and 63 g for breakfast, lunch, and dinner, respectively). The evenly distributed protein intake resulted in 25% higher 24-h fractional mixed muscle MPS vs. the skewed distribution pattern. Assuming a maximal MPS protein intake threshold of about 20 g for this population, the even distribution pattern resulted in 3 meals that met or exceeded the maximal meal protein MPS threshold vs. just one in the skewed distribution pattern. Note that the 90 g total daily protein intake provided in this study amounted to 1.17 g/kg/d, which is considerably more than the RDA. Kim et al. [77] evaluated the effects of protein distribution in older individuals fed diets with total protein at either the RDA or 2X RDA. While they did not observe the same meal distribution effects as Mamerow et al. [76], they did find that protein intake at 2X RDA was associated with greater whole-body net protein balance.

Finally, Norton et al. [78], provided high quality protein supplementation of breakfast and lunch meals to a population of 60 healthy older men and women (mean age 61 y) and compared them with a control group receiving no supplementation. Protein intakes (g/kg) for the supplementation group were 0.4 g/kg for breakfast, 0.5 g/kg for lunch, 0.6 g/kg for dinner, and 0.1 g/kg for snacks, resulting in a total daily protein intake of 1.6 g/kg. By comparison, the control group protein intakes were 0.2 g/kg at breakfast, 0.3 g/kg for lunch, 0.6 g/kg for dinner, and 0.1 g/kg for snacks, for a total daily protein intake of 1.2 g/kg (a figure that already exceeded the protein RDA). The supplemented group, at 24 weeks, had significant increases in both total (0.6 kg) and appendicular (0.28 kg) lean tissue mass. Thus, even compared with subjects having total protein intake that already exceeds the RDA, those that hit a target of at least 0.4 g/kg per meal for the 3 main meals experienced increases in lean tissue mass. These “per meal” protein data support total daily protein recommendations higher than the RDA and, in some populations, emphasis on achieving meal protein intake targets for maximizing MPS and lean tissue mass.

## 7. Do Individual Amino Acid Requirements for Adults Align with Increased IAAO Estimates for Total Protein?

Initial guidelines in the United States for individual indispensable amino acids were published in the 10th edition of the RDAs in 1989 [79] and were based on the information from the Food and Agriculture Organization of the United Nations (FAO) in 1985 [80]. These were re-addressed in the 2005 edition of the DRI [3] and incorporated evidence from nitrogen balance, plasma amino acid concentrations, direct amino acid oxidation (DAAO), 24-h amino acid balance and indirect amino acid oxidation (IAAO) studies.

In accordance with the results of higher published estimated protein requirements demonstrated by the IAAO studies shown in Table 1, there are also multiple IAAO studies of individual amino acid requirements that suggest the RDA is too low. In a population of 16 healthy older adults (mean age 70.4 y), Szweiga et al. [81] reported that the mean leucine requirement was 78.5 mg/kg/d (upper 95% CI 81 mg/kg/d). These mean and upper 95% CI estimations are over double their respective 2005 DRI values (34 mg/kg/d and 42 mg/kg/d). In a young Chinese adult population (mean age 23.7 y) [82], the mean and upper 95% CI lysine estimations were 58.4 and 70.1 mg/kg/d, which exceeded their respective EAR and RDA values by 88% and 84%.

## 8. Physiological Roles and Functions of Dispensable Amino Acids Support the Need for Higher Protein Requirements

An additional advantage of a protein intake recommendation above the RDA would be to help ensure the adequate intake of both indispensable amino acids and “dispensable” (i.e., nonessential amino acids, NEAA) amino acids for which there might be a consistent dietary requirement. Plants and some bacteria can synthesize all amino acids, while mammals, including humans, can only produce 11 and must consume the other 9 essential amino acids from their diet. The National Academy of Medicine (formerly the U.S. Institute of Medicine) classifies these 9 amino acids as indispensable (histidine, isoleucine, leucine, lysine, methionine, phenylalanine, threonine, tryptophan, and valine), and of the 11 that can be synthesized, 5 as dispensable (alanine, aspartic acid, asparagine, glutamic acid, and serine), and 6 as conditionally indispensable (arginine, cysteine, glutamine, glycine, proline, and tyrosine) [3]. In general, indispensable and dispensable amino acids have been differentiated based on two criteria: (1) the ensuing negative nitrogen balance when inadequate levels of indispensable, but not dispensable, amino acids are fed [83] and (2) the ability of the body to synthesize the carbon skeletons of the dispensable amino acids. The first criterion has been problematic for amino acids like histidine and arginine, as described by Hou et al. [84]. In the case of histidine, early studies indicated that feeding histidine-free diets for 8 days did not result in negative nitrogen balance, leading to the erroneous conclusion that there was not a dietary requirement for histidine [83,85]. An explanation for this is that the breakdown of histidine-containing dipeptides such as carnosine in tissues could supply enough histidine over the short-term to maintain nitrogen balance and there would be no net nitrogen loss in this process. Longer term studies restricting histidine have corrected this misperception. Regarding arginine, intakes of arginine when low enough to drastically lower sperm counts and motility, did not result in negative nitrogen balance [83,86]. Either the resumption of a normal diet or the supplementation of arginine returned sperm counts and motility essentially to normal. These data argue that the arginine requirement is not simply related to protein synthesis, but other metabolic functions place a demand on this amino acid. This may well be the case for other amino acids that are precursors to neurotransmitters such as tryptophan and glutamic acid and have other functional roles. Therefore, an understanding of the requirements for each amino acid requires consideration of all its functional roles.

The second criterion is concerning because the ability of the body to synthesize the carbon skeleton of a dispensable amino acid may be misinterpreted to mean that the amount of amino acid synthesized will be adequate to meet requirements. Of the NEAA, only tyrosine is synthesized from an essential amino acid phenylalanine. The other NEAA are formed from either intermediates of glycolysis or the Krebs cycle. Though the carbon backbone originates from a carbohydrate source, these intermediates undergo transamination to produce the dispensable amino acids. The contributed amino group is limited to dietary fixed nitrogen generally contributed from the dispensable amino acids. In its strictest sense, the concept of “dispensable” amino acids would mean consuming only the RDA for indispensable amino acids (~0.2 g/kg/d) [87] would be sufficient to meet protein requirements. However, the RDA for total protein (0.8 g/kg/d) is 4 times higher than the RDA for indispensable amino acids. Thus, it stands to reason that there must be some dietary requirement for dispensable amino acids. Cooper et al. [88] fed in a human study, a diet containing indispensable amino acids at a level of 0.39 g/kg/d, which was higher than the present RDA of 0.2 g/kg/d. Then they added individual dispensable amino acids, one at a time, to boost total protein intake to 0.73 g/kg/d. In this scenario, alanine, arginine, aspartic acid, asparagine, glutamate, glycine, and serine each reduced IAAO relative to the baseline diet containing only the indispensable amino acids. A main conclusion from that paper was that most dispensable amino acids are ideal nitrogen sources for protein synthesis in the presence of adequate indispensable amino acids.

In rats, Heger et al. [89] tested EAA to total protein ratios and showed increased nitrogen retention and utilization from 30% to 80% essential amino acids. At or above 80% essential amino acids, nitrogen retention and utilization decreased. It should be noted that at the 50–80% level of EAA the utilization of EAA decreased, but NEAA utilization increased. This compensatory effect was also seen in pigs [90], which are a more similar model for human digestion, indicating similar results might be found if replicated in humans. The shift in EAA utilization between the 50–80% ratio indicates a metabolic shift is taking place and an ideal ratio lies within this range of EAA: NEAA. The important message is that EAA cannot entirely replace NEAA as a source of nitrogen. Hou et al. [84] have comprehensively reviewed the literature and argue that “there are no compelling data to substantiate the assumption that NEAA are synthesized sufficiently in animals and humans to meet the needs for maximal growth and optimal health”. Hou et al. [91] also noted many physiologically necessary functions performed by dispensable/nonessential amino acids that cannot be accomplished by indispensable/essential amino acids. Compiling data from studies in pigs, poultry, fish, and humans, these authors also noted many instances in which endogenous biosynthesis of dispensable amino acids is not adequate to meet requirements. Thus, they proposed that the NEAA concept for animals or humans is not physiologically valid.

A prominent example among the dispensable/conditionally indispensable amino acids for which synthesis is not likely to meet requirements is glycine. Glycine plays many important physiological roles, including collagen formation (glycine located at every third position in collagen), neurotransmitter function, glutathione production (glycine is one of the amino acids in the glutathione tripeptide), regulation of immune function, and synthesis of other biomolecules including DNA, RNA, creatine, serine, and heme [92]. Gibson et al. [93] studied the effects of a reduction in habitual protein intake from 1.13 g/kg/d to 0.75 g/kg/d on phenylalanine, glycine, and tyrosine kinetics. They reported that glycine synthesis was maintained on the marginal protein diet, but this maintenance required potentially detrimental metabolic adaptations, including reductions in whole-body protein turnover, net protein catabolism, and rate of nitrogen excretion compared with the habitual diet. In an elegant review, Meléndez-Hevia et al. [94] estimated that the combination of glycine available from diet (~3 g/d from the habitual diet in Gibson et al. [93]) and glycine biosynthesis from all known sources (3 g/d) was considerably short of the 14.5 g glycine estimated to be required per day (1.5 g/d for synthesis of metabolites, 12 g/d for collagen synthesis, and 1 g/d for synthesis of other proteins). Thus, even the relatively high protein intake of 1.13 g/kg/d, supplying about 3 g glycine/d, coupled with glycine from biosynthesis, is unlikely to meet biological requirements for optimal function. In concordance, an IAAO study showed that glycine intakes less than 37 mg/kg/d result in increased phenylalanine oxidation among late gestation pregnant women [95] (see next section). Arguments for glycine supplementation have also been made based on the observation that tissue glycine levels are often lower than the glutathione synthase Michaelis constant (K_m_) for glycine [96].

## 9. Support for Higher Protein Recommendations for Older Adults (≥65 Years) Based on Other Nutrition Guidelines

It appears that multiple lines of evidence (IAAO studies, functional benefit studies, studies on potential dietary requirements for NEAA) are converging and pointing to protein requirements higher than the RDA for improved health. Numerous researchers [97,98,99,100,101] have proposed that the RDA for protein is likely too low for older individuals and scientific expert groups [14,102] have recommended protein intakes for older adults of at least 1.0–1.2 g/kg/d, with higher levels (1.2 to 1.5 g/kg/d or more) in situations such as malnutrition, acute and chronic illness, severe illness or injury, and daily vigorous physical activity. In support of the concept of protein requirements above the RDA, Wolfe et al. [1] took an alternative approach, applying the National Academy of Medicine’s Acceptable Macronutrient Distribution Ranges (AMDR), which state acceptable protein intakes of 10–35% of energy, to determination of protein requirements. Consider an example of a 30-year-old male with light activity, a weight of 81 kg (178 lbs.) and height of 1.8 m (5 feet, 11 inches). The estimated total energy expenditure and the daily protein RDA for this individual is 2884 kcal and 64.8 g (0.8 g/kg, or 9% of energy from protein) [3]. The protein AMDR spans from 72.1 g/d (0.91 g/kg) at the low end of the range to 252 g/d (3.11 g/kg). Thus, even the low end of the protein AMDR exceeds the RDA for this individual by 13%.

As per Wolfe et al. [1], daily protein needs can also be estimated via the MyPlate.gov tool. For the 30-year-old male mentioned above, the recommended daily number of servings from each food group [103], and respective protein contents [104], are: 2 cup-equivalents fruit (1 g protein), 3.5 cup-equivalents vegetables (7 g protein), 9 oz.-equivalents grains (27 g protein), 6.5 oz.-equivalents protein (45.5 g protein), and 3 cup-equivalents dairy (24 g protein). The estimated total protein intake in this scenario would be 104.5 g/d, or 1.29 g/kg (16% dietary energy), which is, again, higher than the RDA. Given that data from the National Health and Nutrition Examination Survey suggest that protein intakes in U.S. adults are 14–16% of energy [105], the protein intake recommendations suggested by either the AMDR or MyPlate.gov agree closely with self-selected protein intakes in the population. As such, it appears that the RDA is an outlier on the low side compared with these other approaches. Although there is growing recognition of the value of protein intakes greater than the RDA for health benefits in the older adult population, there has been little mention in the scientific literature of promoting similar higher protein intakes for younger adults or other populations. Clearly, the abundance of evidence in favor of protein intakes above the RDA has been collected in older adults. As shown in Table 1, there is some evidence from IAAO studies for increased protein requirements in other populations such as children, young adults, athletes, and pregnant women. In the next section of this paper, there will be a focus on protein intake recommendations for pregnant and lactating women and children.

## 10. Determination of Protein Recommendations for the Pregnant and Breastfeeding Populations

Pregnant women represent one particularly important population when considering both the ramifications of new estimates of protein requirements from IAAO studies and the potential importance of a wisely planned diet for pregnancy. Adjustments in protein metabolism occur within the first several weeks of pregnancy. Greater energy and protein demand is not distributed evenly throughout the pregnancy, but gradually increases at each trimester. The incrementally greater requirements of late pregnancy must be met partly by physiologic adjustments in nitrogen metabolism induced by pregnancy and partly by increased dietary intake as there is no evidence that pregnant women store protein early in gestation for later fetal demands [106].

In determining the protein requirements for the pregnant and lactating population, calculations must integrate a component for protein homeostasis or maintenance for mother, but also factor in growth or tissue accretion of the infant. The WHO defines adequacy roughly as the lowest protein intake to balance losses of nitrogen from the body from a person at energy balance in pregnancy and lactation, while meeting the additional needs associated with fetal growth and milk production consistent with “good health” [4]. Adequacy for the average of this population, the EAR from the 2005 DRI is 0.88 g/kg/d, which factors in 0.22 g/kg/d for growth and 0.66 g/kg/d to account for protein maintenance needs [3]. With the addition of 2 times the coefficient of variation, the calculated RDA for approximately 98% of the pregnant population is 1.1 g/kg/d [3]. For lactation the calculated protein needs are greater to sustain growth of the infant minimally through the first 6 months of life. The EAR is 1.05 g/kg/d and the RDA is 1.3 g/kg/d [3]. There are several reasons why these numbers should be reexamined for this population. These nitrogen balance calculations were based on young men, not the pregnant or lactating population, when the linear regression analysis was originally applied to this data, it did not include the data points at higher protein intake as mentioned previously [20], and whole protein turnover is increased in the latter two trimesters indicating potentially higher protein demand and need for an incrementally greater EAR and RDA for protein in late gestation [107].

The rate of protein accretion in pregnancy has been measured directly through nitrogen balance and indirectly from an increase in whole body potassium. Both methods indicate higher nitrogen retention compared to gains calculated by the factorial method. In the 2005 DRI, it was estimated (as a mean of the last 2 trimesters) that the daily amount of additional protein to support maternal weight gain was 8.4 g/d and the amount to support protein deposition in the fetus was 12.6 g/d (total of +21 g/d). So, one approach to estimating the EAR was to simply add 21 g/d to the nonpregnancy protein EAR of 0.66 g/kg/d. For a hypothetical woman with a pregravid weight of 57 kg, this translates to an EAR of 58.6 g/d. An alternative approach was to simply multiply the woman’s body weight by 0.88 g/kg/d, resulting in an EAR for the hypothetical 57-kg pregnant woman that could range from 50.2 g/d at the start of gestation to 64.2 g/d at the end of gestation (assuming a pregnancy weight gain of 16 kg). In an older observational study, Burke et al. [108] reported low birth weight and short length for age in infants born to mothers averaging less than 75 g of protein intake per day. This is a timepoint predating the obesity epidemic when the average weight of the American population was smaller. In addition, Higgins et al. [109] showed increasing the daily protein intake in pregnant low-income women to 101 g/d on average helped improve birth outcomes. These supplemental food trials considered during the development of the DRIs imply a much higher daily protein intake supports better birth outcomes [3].

## 11. Support for Higher Protein Recommendations for the Pregnant and Breastfeeding Populations

Most contemporary studies measuring protein intake during pregnancy support these early findings. A recent observational study in Spain showed even a minor increase in daily protein in well-nourished women was associated with a significant increase in birthweight for 25th (67.7–71.5 g/d), 50th (76.4–80.5 g/d), and 75th (86–93 g/d) percentile groups from preconception until 38 weeks of pregnancy. The researcher tracked all macronutrient intakes and participants increased or decreased all macronutrients uniformly over timepoints indicating a balanced change or a maintenance of macronutrient distribution is important for healthy birth outcomes [110].

Similarly, a Cochrane review of 13 randomized trials reported that balanced energy/protein supplementation was associated with increased birth weight and substantial reduction in risk for small for gestational age but did not show positive effects in many other birth outcomes [111]. Only 1 of the Cochrane review studies shared estimated daily protein intake, which amounted to 30–40 g protein per day for a 2000 kcal diet [112]. Imdad and Bhutta [113] support this recommendation of balanced energy/protein supplementation with protein <25% of energy in a review indicating that such supplementation was associated with a significant 31% reduction in the risk of giving birth to small for gestational age infants, and this effect was more pronounced in undernourished women. The protein intake ranges were not shared in this publication but for a 2000 kcal diet would be estimated as 50–125 g/day. Another longitudinal study examined prenatal and postnatal macronutrient intake and impact to offspring macronutrient intake and body composition later in childhood (9–11 years). When adjustments were made for underreporting and energy, prenatal macronutrient and energy intake was not associated with offspring fat or lean mass and all maternal macronutrient intakes whether prenatal or postnatal were positively associated with intakes of the same nutrients in offspring, indicating role-modeling has the lasting pattern on offspring eating patterns [114]. Currently, conclusions on high protein supplementation are based on one study from 1980 with a low-income cohort likely not receiving adequate prenatal care and with a history of LBW infants. In this study, balanced protein calorie supplementation compared to high protein supplementation did improve outcomes (improved length at gestation, decrease in LBW, and increase in mean birthweight) [115]. The intervention groups with supplement achieved close to 2400 kcal per day and the high protein group in this study consumed approximately 101 g/day protein, the balanced protein/calorie group 86 g/day, and the control group 79 g/day per 2000 kcal. Lack of documentation regarding protein intake, lack of standardization regarding what is considered high vs. low, and the poor study design of studies incorporated in the Cochrane review make comparative conclusions impossible other than that protein intake in the context of balanced macronutrient delivery even if greater than the RDA has proven to support the healthiest outcomes.

Conversely, other recent observational analyses are mixed on the relationship of increase of protein intake during pregnancy and outcomes, such as infant size and body composition at birth. The prospective observational Healthy Start Study indicated infant adiposity, but not birthweight, is independently associated with increased maternal intake of total fats, and total carbohydrates, but not protein. Protein intake was not associated with FFM or birthweight [116]. A large longitudinal study of pregnant women that included both women who were enrolled in Women Infants and Children (WIC) and women who were not WIC eligible compared neonatal birthweight in a low protein intake group (<50 g/d), an intermediate group (50–84.0 g/d), and a high protein intake group (≥85 g/d). When controlling for covariates, Sloan et al. reported birth weights for the low protein group had decreased by a mean of 77 g and the high protein group had decreased by a mean of 71 g compared to the intermediate group [117]. Morisaki et al. (2018) in a very large observational study indicated an inverse u-shaped relationship with protein intake and effect on birthweight [118]. Switkowski et al. [119] examined the relationship between protein intake during pregnancy and neonatal length and reported a mean protein intake of 1.4 g/kg/d in 1st and 2nd trimesters was associated with −0.1 change in length Z-score but did not examine intake in 3rd trimester the period of greatest nitrogen accretion during pregnancy. Several covariates were adjusted for, except for total energy intake and maternal BMI [119]. This is an important limitation to recognize as adjustment for total energy is likely the most important factor in detecting a potential positive effect of protein supplementation. These studies do not dismiss a potential greater demand for total protein during pregnancy, but more likely indicate the need for better controls in study design, especially monitoring infant body composition based on intervention, and an incremental increase in protein (which is not an isocaloric swap for calories) over the course of gestation beyond just the calculated increase due to maternal weight gain. It is suggested that inconsistencies from previous studies on the relationship of maternal dietary intake and neonatal anthropometrics or body composition may be explained, in part, by error in adjustment for energy intake associated with the FFQ tool used, inherent underreporting of dietary intake on FFQs, especially in individuals with higher BMIs and higher daily energy intakes [116]. In addition, higher maternal diet quality according to the Healthy Eating Index-2015 measured prenatally through 3 months post-partum in a prospectively followed cohort from the Mothers and Infants LinKed for Health (MILK) study was associated with lower infant weight for length *Z*-score (WLZ) and infant percent body fat from birth to six months, but not length-for-age *Z*-score (LAZ) or weight-for-age *Z*-score (WAZ) [120]. This evidence signifies the importance of not using one metric such as infant birth weight as an indicator of the health of maternal diet.

For a diet of 2500 kcal, 10–25% of energy would prescribe a range of 62.5–156 g protein per day and for the 57 kg woman the calculated g/kg/d would amount to 1.1–2.7 g/kg/d. The lowest percent in the recommended macronutrient distribution provides the equivalent of the current RDA with all other percentages exceeding it. Morisaki et al. reported lowest risk for SGA and greatest effect on birthweight at a protein density of 13% of energy. For the 57 kg women this would amount to 81.3 g of protein per day and 1.4 g/kg/d and categorize this woman in the intermediate protein group described by Sloan et al. with the highest birthweights. This mathematical exercise illustrates simply that the RDA is the lowest prescribed intake according to the AMDRs. In addition, the breadth of the positive birth outcomes fell within healthy, balanced protein densities greater than the RDA.

## 12. Support for Higher Protein Recommendations for the Pregnant and Breastfeeding Populations: IAAO Studies

The IAAO method was used to determine the protein requirement for two time points during singleton pregnancy; early in gestation (16 weeks; 1.2 g/kg/d) and later in gestation (36 weeks; 1.52 g/kg/d) [38]. Calculated protein requirements for both timepoints were greater than the current RDA. These values coincide with the data from Higgins et al. [109] mentioned above (101 g/d or 1.38 g/kg/d) and also intake values of healthy pregnant women in a prospective study in Vancouver, Canada (incidence of LBW is low) where the median range of protein intake was 1.3–1/5 g/kg/d for early (16 weeks) and later (36 weeks) gestation [121]. The IAAO protein requirements in the context of total calories represent 14–17% of calories. The important point that emerges from these data is that the estimated protein needs for the pregnant population exceed the current RDA by 15–27%. Also, the estimated protein needs were not static through the course of pregnancy, with a greater increase in requirement being reserved for the latter part of gestation. This evidence supports the concept of a specified protein requirement per trimester of pregnancy.

The increased protein requirements as calculated by the IAAO method for pregnancy bring into question the RDA for lactation, which puts even greater protein and caloric demands on the mother. The estimated protein needs are based on the average daily output of non-protein nitrogen and protein in breastmilk multiplied by a factor of protein utilization efficiency added to the 0.66 g/kg/d extrapolated from the young male population. The current EAR and RDA for lactation are 1.05 g/kg/d and 1.3 g/kg/d respectively. With the knowledge that lactation increases nutritional demand, it is logical to question how the EAR 0.66 g/kg recommendation for protein intake measured in young sedentary men could match the incremental needs that milk production places on the human body. This calculation does not embrace that the production of breastmilk, an extraordinarily complex matrix, requires an incremental demand for protein beyond that of maintenance for the mother, and should account for the additional metabolic processes of milk production which includes the increased mass of mammary glands to support production. In addition, these calculations from the 2005 DRI were based on the average reference weight of 14–18 years-old women, 54 kg [3]. Women in developed countries are having children much later in life (mean age 30 years) [122] and on average exceed the 54 kg weight used to determine needs. According to the CDC, the average weight of an American woman greater than 20 years is 170.8 lbs. or 76.6 kg [123]. Estimations of the average protein requirements for women exclusively breastfeeding 3–6 months postpartum using the IAAO method ranged from 1.7–1.9 g/kg/d, 61–81% higher than the EAR [39]. The large discrepancy between the RDA and the IAAO estimate may possibly be explained by multiple factors which could include the use of a less representative figure for protein maintenance (the value derived for sedentary young men 0.66 g/kg/d) demand in calculating nitrogen requirements for a breastfeeding woman and the women used in the IAAO study likely had a larger average weight. Regardless, the estimate implies an even greater nitrogen demand for lactation compared to the RDA.

## 13. New Estimates for Individual Amino Acid Requirements for the Pregnant Population, How They Compare with Increased IAAO Estimates for Total Protein and Evidence for Setting Specific Requirements for Each Trimester

The demand for the indispensable amino acids is greater for the pregnant population as compared to the general adult population. The EAR for the indispensable amino acids for this population was calculated using a factorial approach by multiplying the estimate for adults by a factor of 1.33 (to account for fetal accretion) as was done when calculating additional needs for total protein for this population, with no difference recommended for the stages of gestation. However, the indispensable amino acid requirements increase differentially in later pregnancy as compared with early pregnancy in pig models: threonine by 55%, lysine by 45%, isoleucine by 63%, and tryptophan by 35% [124,125,126,127]. IAAO analysis of lysine requirements in the pregnant population revealed an increased proposed EAR for late pregnancy (50.3 mg/kg/day), but the proposed EAR requirement for early pregnancy (36.6 mg/kg/d) was comparable to the level as measured previously for the adult population (36 mg/kg/d). The proposed requirement for early gestation is less than the current EAR for pregnancy (41 mg/kg/d); however, the late gestation value is 23% higher than the recommended EAR for lysine [128]. This data indicates the possibility of a need for separate requirements for individual amino acids during the different trimesters of pregnancy and aligns with IAAO estimates for total protein requirements for the pregnant population.

The same group of researchers tested the requirements for the total aromatic amino acids (TAA) (in the absence of tyrosine), in early and late gestation using the IAAO method [129]. Increased requirements for TAA were estimated compared with the existing RDA in early and late gestation. In early gestation, the measured TAA requirement was 44 mg/kg/d (95% CI 28.3, 58.8) and in late gestation it was 50 mg/kg/d (95% CI 36.1, 63.1). If the upper bound of each of these 95% confidence intervals is taken to represent a population safe intake, interpreted similarly to the RDA, then these estimations represent increases of 34% in early gestation and a 43% increase in late gestation compared with the RDA (44 mg/kg/d) for the TAA.

The results for TAA were not entirely similar to those observed for lysine, where requirements did not increase early in gestation. It should be noted that the DRI values for the indispensable amino acids rely on a small set of data points, some with results with large ranges in value. The data supporting the current TAA ranged from 15.1 to 39 with an average of 27, while lysine had the most data points and ranged from 26.6–36.9 mg/kg/d [3]. Measures for the TAA and lysine that were determined from the IAAO correlate better with the higher estimates determined in the nitrogen balance studies performed previously and used to support the values in the DRI.

There is no EAR for each individual dispensable amino acid for any population. Yet, swine studies showed greater demand during pregnancy for the two dispensable amino acids glutamine and arginine, suggesting they were conditionally essential [130]. Rasmussen et al. [95], tested glycine, a dispensable amino acid, using IAAO in the pregnant population during mid- and late gestation. During mid-gestation (~26 wks), a glycine-restricted diet did not affect whole-body protein synthesis, suggesting de novo glycine synthesis from interconversion of other amino acids such as serine was adequate for the maternal needs [95]. However, in late gestation, the restriction of glycine did impact whole body protein synthesis. The researchers were able to calculate a breakpoint (>37 mg/kg/d) using 2-phase linear regression indicating limiting glycine restricted protein synthesis in late gestation. Other methodologies to estimate daily glycine requirements for a population safe estimate suggest levels between 46–59 mg/kg/d in healthy adults [131]. This would suggest the requirement for late gestation should be higher than these estimates. Rasmussen et al. [95], advised the lower calculated value (>37 mg/kg/d) may be due to provision of total protein at 0.88 g/kg/d during the test. Inadequate provision of total nitrogen, which at 0.88 g/kg/d is the EAR, not the population-safe level of protein, may have impacted the de novo synthesis of glycine in later gestation when protein needs are higher. Therefore, provision of adequate total protein would likely have resulted in a higher glycine breakpoint in late gestation.

Radio-labeled tracer tests to evaluate dispensable amino acid demand during mid- and late gestation also support higher requirements during the later stage of pregnancy. Endogenous glycine and arginine flux, which is derived from protein breakdown and de novo synthesis, was determined for pregnant adolescents compared to adults. In adolescent pregnancy when demands for nitrogen are compounded by maternal growth demands for protein, Thame et al. [132] found that the arginine flux decreased in the 3rd trimester in the adolescents, but not in the adult pregnant women. This indicates the adolescents could not maintain arginine production to meet the increased demands of later pregnancy combined with their own needs for growth. This difference was not evident in the first trimester. Thame et al. [133] also reported a 39% drop in glycine flux in the adolescents and a 5% increase in the adult group from trimester 1 to 3. Again, the adolescent population could not maintain glycine production during mid-pregnancy [133]. In this well-nourished population, there was no marked negative effect on pregnancy outcome and there were no significant differences in baby’s birth weight and length between the two groups. However, there was an observable trend with the adolescent group’s baby’s birth weight and length being 8.6% and 6% less, respectively, compared with the mean values of the infants born to the adult group. As outlined earlier, glycine participates in many physiological functions and makes up a third of the amino acids in collagen, which is the most abundant protein in mammals, making up about 25% to 35% of the whole-body protein content [134]. Therefore, it is possible that during a time of such intense growth velocity that the most prevalent amino acid, glycine, in the most abundant body protein could play a role in growth rates and possibly growth restriction. Kurpad et al. [135] observed a decrease in glycine flux in both groups in a study with low BMI pregnant and normal BMI pregnant women from trimester 1 to 2. These authors did not measure glycine flux in the 3rd trimester. Had the study captured differences between trimester 1 and 3, there may have been a greater decrease in glycine flux as collagen synthesis comprises a large percent of fetal protein accretion and fetal membrane development in late gestation. These experiments are difficult to perform as the proteinogenic demand for a single dispensable amino acid which is involved in many other biochemical processes is not easily isolated. They do, though provide two valuable insights: (1) protein requirements in the latter stages of pregnancy are greater, confirmed at least in the case of the adolescent who has higher nitrogen demands and (2) when intake is limiting to a point approaching a threshold likely closer to demand, these experiments are sensitive enough to reveal a greater conditional demand for some dispensable amino acids during pregnancy. Cumulatively, these tests on individual amino acids support higher requirements for the later part of gestation and align with total protein estimates performed by IAAO.

## 14. Support for Higher Protein Recommendations for Healthy Children to Support Growth

The current RDA recommendations for protein in children set at 1.1 g/kg/day for 1–3 years, 0.95 g/kg/day for 4–8 years and 9–13 years, 0.85 g/kg/day for 14–18 years according to the latest DRI in 2002/2005 [3]. The requirement set for ages 1–3 specifies the largest gram per kilogram weight value as this is the period with the fastest growth trajectory. The grams per kg. weight as set begin to diminish at the age of onset of puberty, the second fastest period of growth, yet during this second largest growth spurt adolescents will amass 15–20% of adult height and 50% of adult body weight [136]. The current protein recommendations are based on a factorial estimate. This method takes the daily protein requirement measured by nitrogen balance studies in adults, which estimates needs for maintenance and adds a factor intended to estimate additional nitrogen needs for accretion or growth. It additionally considers the inefficiency of protein utilization in children as compared to adults. Gattas et al. [137] performed one of the few studies that measured nitrogen balance directly in this population. The derived population safe estimate for healthy boys, 8–10 years old was 1.2 g/kg/d [137]. Other recent stable isotope studies challenge the calculated RDA for children 4–13 years which may be an underestimation of needs by as much as 60% [138]. It should be noted also that the RDA is not graded for different intensities of activity. Young athletes may create an additional nutritional burden (e.g., development of greater muscle mass) which would potentially require an increase in protein. Measures of the rates of muscle protein turnover in children in response to acute exercise are not available. However, two groups reported decrease in protein turnover in young boys and girls (8–10 years) who were either walking (protein 15% of calories and 2 times the RDA, >2 g/kg/d) or participating in a resistance exercise program (protein 16% of calories and >1.5 g/kg/d). Children were their own control and, diets and protein intake did not change baseline to post-observation. These turnover experiments were conducted after a 10-h overnight fast with labeled tracer. This indicates a shift in utilization of amino acids and brings into question whether provision of protein was adequate during these short-term studies [139,140] and whether the current RDA specifies adequate protein for growth and may fall even shorter in cases of intense physical exercise. This might necessitate an additional set of protein requirements for child athletes which would provide an appropriate protein supply to deliver enough protein for both normal muscle growth and maximal growth.

## 15. Support for Higher Protein Recommendations for Healthy Children: IAAO Studies

Elango et al. [30] performed direct measurement of nitrogen utilization by the minimally invasive technique IAAO in children 6–10 years, which indicated an EAR at 1.3 g/kg/day and an RDA of 1.55 g/kg/day protein. The current RDA for this age group is 39% lower than the estimation from Elango et al. [30]. Currently IAAO studies have not been performed with undernourished adults or children to provide an insight into potential differences in protein utilization and efficiency.

## 16. Do Individual Amino Acid Requirements for Healthy Children Align with Increased IAAO Estimates for Total Protein?

The efficiency of protein utilization can depend upon several factors relating to the metabolism of the individual, the digestibility of the protein, absorption and transport of the individual amino acids. IAAO studies have now been employed to determine if the utilization of each individual amino acid was similar for adult and child to test the applicability of the factorial approach in assigning protein maintenance requirements for children. The results of experiments to test the requirements for individual amino acids using the IAAO method have been mixed.

Table 3 enables a side-by-side comparison of several proposed IAAO estimates for individual amino acids that were measured in both the adult and child populations. Values for the adult population safe estimates align well with values set in the last DRI with the exceptions of marginal increases in demand for lysine and the TAAs and a significant increase in demand for the BCAA (2.1 times the value set in the DRI) which could largely account for the greater proposed RDA for total protein for adults reported by Elango et al. 2012 [3,29]. Four of the 5 estimates for children (Lysine [141,142], BCAA [143], SAA [144], and TAA [145]) are lower than the estimates for adults and 2 are lower than the current DRIs for their age range and adults (TAA [146] and SAA [147]. Hsu et al. [146] describe the IAAO value derived for TAA by IAAO as being “biologically absurd” as nitrogen balance studies have demonstrated young, growing children require more protein and essential amino acids for purposes of nitrogen accretion. It is hypothesized the lower IAAO value may be explained by a lower rate of hydroxylation of phenylalanine by children [146]. Variability of urinary values of these amino acids indicated interindividual difference in hydroxylation rates with lower values in children indicating even lower enzyme efficiency rates. The IAAO values for the total SAAs were not as low as that evaluated for TAA for children. Like the TAA, the essential amino acid in this case is required for production of a non-essential amino acid. Cysteine is not essential when adequate serine and methionine as the sulfur donor are available. The interdependency of these reactions and others that these substrates are involved in and the enzymatic efficiency in children compared to adults could contribute to the lower derived values. When this process was employed for the branched chain amino acids (BCAA) which make up 14% of the amino acids in skeletal muscle, the RDA was 48% higher [130] than the current DRI recommendations for the total BCAA intake for school age children [3]. Interestingly, the IAAO requirement for BCAA healthy men [148] was 9% higher than for school age children [143]. Two values are indicated for lysine in Table 3. An additional lysine requirement study was performed on healthy Indian children to determine if children who generally consume a more cereal focused diet, which is lower in lysine may have developed metabolic adaptations in utilization of this essential amino acid. The EARs were nearly identical to each other (33.5 and 35 mg/kg/d for the Indian and Canadian children respectively) and the current EAR requirement 37 mg/kg/d [142]. The calculated RDA for Indian children was considerably lower than that calculated for the Canadian children, but equivalent to the current RDA [142]. In an identical study, testing undernourished Indian children before and after treatment for intestinal parasitic infestation the authors showed the infestation increased requirements for lysine by 20% [149]. Only the estimation for BCAAs in children greatly exceeded their current RDA. The minor differences seen between the measures for adults compared to children suggest they may both represent a maintenance number for nitrogen balance and studies over a longer timeframe may be required to capture the incremental increase that growth demand places on protein synthesis. This supports the use of the factorial approach whereby an additional factor related to efficiency of nitrogen utilization and accretion should be added to the value determined by IAAO for the individual amino acids [144]. Measures that include multiple amino acids where one is a precursor to the other, such as the TAA and SAA may be complicated by other factors related to enzyme kinetics. Slower enzymatic conversion rates of the enzymes in these reactions may be confounding the results whereby the limiting amino acid oxidation rate plateaus prematurely and underestimates the specific needs of a growing child. Thus far the IAAO studies have reported only increased needs for children regarding total protein and for the BCAAs and in instances in parasitic infestation.

## 17. The Influence of Protein Quality on the Need for Higher Protein and Amino Acid Recommendations for Undernourished Children Based on Other Study Data

Provision of protein and amino acids should be considered not only in the context of the obvious role of supply of nitrogen for protein synthesis, but also in the context of the role specific amino acids play in stimulating growth. This is most visible as it relates to the issue of stunting. Statistically, in 2014 it was estimated that ¼ of the population of children under 5 years of age were stunted [153]. Stunting, related to chronic malnutrition, an annual 2.1 trillion USD global, public health threat [154] has a life-long impact on the children afflicted by it; an increased risk of child mortality, infectious disease morbidity, impaired neurocognitive development, and metabolic diseases [155]. The focus on the etiology of malnutrition has shifted over the decades from protein to micronutrient deficiency in the belief that most children consume enough dietary protein. Semba et al. [156] addresses this assumption and the need to re-examine protein adequacy in terms of specific amino acids. The study used a targeted metabolomic approach in a population of young children in Malawi, to measure serum amino acids and other select metabolites such as glycerophospholipids and sphingolipids. Metabolic changes were observed in the stunted children; lower serum concentrations of all nine essential amino acids (tryptophan, isoleucine, leucine, valine, methionine, threonine, histidine, phenylalanine, lysine), significantly lower serum concentrations of conditionally essential amino acids (arginine, glycine, glutamine), non-essential amino acids (asparagine, glutamate, serine), six different sphingolipids, and alterations in serum glycerophospholipid concentrations compared with non-stunted children [156]. These notable differences indicate that lower intake of most amino acids was a potential contributing factor in stunting and may not be solely attributed to micronutrient deficiencies.

A review by Arsenault and Brown [155] examined growth studies in children through the lens of supplementation of higher protein and studies that fortified poorer quality protein sources with individual amino acids such as lysine, which is low in cereal-based diets. Eighteen fit the criteria. In 8 studies, the cohorts were hospitalized children, mostly under the age of 3 years with acute malnutrition and reported normal growth rates with the recommended protein intake levels for healthy children but saw more rapid growth related to weight gain for age and size with higher protein intakes. Ten community-based studies with weaker study designs, such as not controlling for baseline status longitudinally or dietary intake did not support the benefit of more rapid growth with higher protein intakes [155]. Arsenault addresses the fact that these studies are older, not well designed, and not well powered, which limits the emphasis that can be placed on them yet communicates they provide some degree of insight. There is increasing concern for over-fortification of protein in the infant and very young children populations relating to healthy weight gain and growth [157]. Quality over quantity of protein must be addressed in discussion of optimal nutrition. The review by Arsenault examines a few studies performed using a newly discovered maize cultivar named quality protein maize (QPM) with twice the amount of lysine and tryptophan, as well as protein bioavailability similar to milk casein. It was used to replace conventional maize in nutritional studies in undernourished children in Africa and Latin America. A meta-analysis conducted using 9 of these studies found consumption of QPM instead of common maize can significantly improve child growth. The QPM groups showed an average of 12% height increase and 9% weight increase compared to children consuming conventional maize from baseline to end of study [158]. These studies, despite study design flaws, reinforce the concept that when swapping out a protein system with a higher quality, more bioavailable protein one can improve growth outcomes in an intervention study designed to help undernourished children. Protein quality and bioavailability are significant factors which should be considered in determining protein requirements.

In reaction to the reduction of the 2007 FAO/WHO protein requirement estimates for children, Ghosh et al. [17] performed a calculated adjustment of dietary protein (defined as “utilizable” protein) by factoring in quality, digestibility, effect of infection and mild energy deficit. Using data from the food balance sheets (FBS) from the FAO for the year 2005, the research found a negative association between rates of stunting and the per capita availability of “utilizable” protein but when controlling for energy intake, total protein intake was not a statistically significant factor while “utilizable” protein intake was still negatively associated [17]. More recently, a cross-sectional survey in two regions of the Congo showed lower total protein intake (when corrected for digestibility and bioavailability) in combination with low methionine and branched chain amino acids below the EAR in the group of children with greater prevalence of kwashiorkor compared to children living in the area with lower prevalence [159]. It is worth noting that the median intake for all amino acids in these children’s diets exceeded the WHO minimum requirements. This evidence is correlative not causative but indicates a possible relationship between amino acid profile of the diet and not strictly total protein intake and protein malnutrition. It exemplifies an environment where protein requirements appeared to be met yet did not meet an operational definition of protein sufficiency.

In a presentation in the Keystone Symposium, “Optimizing Nutrition for Maternal, Newborn, and Child Health”, Anura Kurpad reported data from a study using the dual isotope method of an intrinsically labeled protein to determine amino acid digestibility in children <2 years who were either malnourished or malnourished and stunted with a length-for-age Z-score (LAZ) of <−2 [160]. The stunted children had slightly lower values of markers for environmental enteric disfunction (EED) with only one marker showing statistical difference, kynurenine: Tryptophan ratio, a marker of inflammation. The digestibility of a few amino acids, the BCAA and methionine (SAA) were 10% lower in stunted compared to non-stunted children [160]. This indicates a metabolic difference that impacts digestibility and absorption, which may or may not be related to EED. Regardless, it is possibly the reduction of availability of essential amino acids that could be driving the stunting between these two groups of undernourished children. It begs the question whether provision of more BCAA and SAA could improve growth outcomes for stunted children.

How to interpret the result of low serum amino acids is not yet clear. Some potential causal candidates are low amino acid pool related to acute protein deficiency, poor digestion and absorption related to gut integrity, presence of infection impairing protein utilization (the immune system partitioning the limited amino acids) or metabolic adjustment which could be transient or permanent. The findings do indicate a need for growth studies to control for variables such as digestibility, infection, and essential amino acid profile in comparison to protein requirements [161]. Cumulatively this evidence, though not perfect in all its design does raise the possibility that higher protein levels (to improve intake of essential amino acids) or potentially increased fortification of specific amino acids may improve growth outcomes in undernourished children. Most compelling is the fact that stunting, and malnutrition induce metabolic differences. Therefore, treating these children could require a special approach and carefully designed efficacy studies to determine optimal total protein and amino acid provision to improve catch up growth.

## 18. Limitations

This review article is not a systematic review or meta-analysis which utilized strict criteria in selection of publications for inclusion. The evidence compiled is comprehensive, but not an exhaustive screening of evidence. Publications were included based on: (1) their historical importance in development of the DRIs and understanding protein requirements; (2) provision of new data on protein requirements since the re-evaluation of the DRI; and (3) if they included interventions that addressed functional health outcomes when greater than the RDA of protein was delivered and whether the outcomes supported or contradicted this practice. It should also be noted that this paper was not intended to be an in-depth review of the limitations of or technical methodologies of the nitrogen balance or amino acid oxidation-based approaches for estimating protein requirements.

Finally, the issue of protein quality is important, but could not be addressed in depth in this paper. Public health organizations such as the IOM and WHO/FAO have not integrated a qualification of protein quality in their determinations of total protein intake recommendations. At present, there is not a globally recognized best practice for estimation of protein quality and digestibility. The FDA and Health Canada still utilize the protein efficiency ratio (PER) study (digestibility and quality as determined by the growth of a young rat). The Food and Agricultural Organization of the United Nations (FAO) and the U.S. National Academy of Sciences use the Protein Digestibility Corrected Amino Acid Score (PDCAAS) where the most limiting amino acid score is corrected for the fecal true digestibility of the protein as determined by fecal digestibility measured in a laboratory animal such as the rat. The FAO introduced the Digestible Indispensable Amino Acid Score (DIAAS) method in 2011 [162]. which is calculated and interpreted similarly to the PDCAAS but uses an updated reference pattern for the indispensable amino acids and an alternative digestibility correction based on ileal amino acid digestibility, as opposed to true fecal nitrogen digestibility that is utilized in the PDCAAS. It should also be noted that the IAAO testing methodologies for estimating protein requirements use a high quality, highly digestible source in their studies. Realistically most individuals even in many developed nations do not consume high quality protein. This is worthy of consideration in development of protein guidelines. Global agreement on best practice to determine protein quality and adoption of a statement on protein quality should impact intake levels or designation of a range of protein requirements which recommend a higher intake where protein quality of a population is low could better guide healthcare professionals.

## 19. Conclusions

As presented throughout this paper, multiple lines of evidence point to a need to reevaluate the protein and individual amino acid requirements for several populations. Disparate results regarding nitrogen requirements may indicate the need to incorporate the results of different methodologies to develop a more holistic picture of the real needs of these populations, including real world data with functional outcomes, nitrogen balance data, IAAO studies, dual isotope studies, and calculations based on other dietary recommendation such as the AMDR and country specific guidelines. Reliance on one methodology such as nitrogen balance studies, especially by restricting the types of studies chosen and limitations on the type of calculations used to interpret the data may be too restrictive to develop a picture of protein needs that is inclusive of all populations, life stages, and health conditions. Historical data and new evidence on health and functional outcomes for adults and the pregnant population generally support higher protein recommendations. IAAO studies indicate children may have higher protein demands as well or higher protein may be required if its quality is not adequate, as evidenced by epidemiological data and studies on protein malnutrition. The functional data on individual amino acids reviewed also reinforces the point that each amino acid whether dispensable or indispensable should be considered as a unique nutrient and calculation of daily requirements may require an alternate estimate of needs for distinctive life stages and populations. Protein requirements in the U.S. have not been evaluated since 2005 and many studies published since then support protein recommendations above the RDA for multiple populations. In addition, expert societies, that published guidance on protein requirements for specific populations are reexamining needs for these populations. The cumulative evidence reviewed warrants an updated analysis of protein requirements by governmental organizations who set dietary policies. Such an analysis should incorporate a wider body of evidence to substantiate the total protein and amino acid needs, as each method used to determine these has limitations, including nitrogen balance determinations.

## Figures and Tables

**Table 1:** Comparison of estimated protein requirements from IAAO studies relative to current recommendations (DRI RDAs or RNI).

Population	Mean Age or Age Range (y)	Proposed EAR (g/kg BW/d)	Current EAR (g/kg BW/d)	Proposed Population Safe Intake (e.g., RDA or RNI, g/kg BW/d)	Current Population Safe Intake (e.g., RDA, RNI, g/kg BW/d)	Reference
Bodybuilders, male	22.5	1.70	0.66	2.20	0.80	[10]
Endurance trained males, 24 h post exercise	26.6	2.10	0.66	2.60	0.80	[11]
Endurance athletes, male	28	1.65	0.66	1.83	0.80	[12]
Resistance-trained females	23	1.49	0.66	1.93	0.80	[13]
Young adult males	~27	0.93	0.66	1.20	0.80	[22]
Children	6–11	1.30	0.76	1.55	0.95	[30]
Young adults, China	21	0.87	0.92	0.98	1.16	[31]
Young female adults, China	21	0.91	0.92	1.09	1.16	[32]
Female athletes, variable intensity exercise	21.2	1.41	0.66	1.71	0.80	[33]
Older males	>65	0.94	0.66	1.24	0.80	[34]
Older females	>65	0.96	0.66	1.29	0.80	[35]
Older adults, China	>65	0.91	0.88	1.17	0.98	[36]
Octogenarian females	82	0.85	0.66	1.15	0.80	[37]
Pregnant women	24–37					[38]
Early gestation (11–20 wks)		1.22	0.88	1.66 (upper end of 95% CI)	1.10	
Late gestation (31–38 wks)		1.52	0.88	1.77 (upper end of 95% CI)	1.10	
Lactating Women (3–6 mo. Postpartum)		1.7–1.9	1.05	NA	1.30	[39]

EAR = Estimated Average Requirement; DRI = Dietary Reference Intakes, RDA = Recommended Dietary Allowances; RNI = Recommend Nutrient Intake, NA = Not Available. Requirements/recommendations stated in g protein intake/kg BW/d. To convert recommendations to g/lb. BW/d, divide the above numbers by 2.2.

**Table 2:** Studies reporting different health or functional/performance benefits of higher vs. lower protein intakes.

Study	Population (*n*)	Study Details	Results
Bartali et al., 2012 [47]	Community-dwelling men and women ≥ 65 y (598)	Mean protein intake 77 g/d (48.5 g animal protein/d); mean energy intake 1999 kcal/d	Main effect of protein on muscle strength was not significant Lower protein intake was associated with greater decline in muscle strength in those with higher inflammatory markers (CRP *, IL-6 *, TNF-α *)
Beasley et al., 2013 [48]	Postmenopausal women 65–79 y (5346)	Calibrated energy and protein intake and physical function assessed	Calibrated protein intake in quintile 5 (15–22.3% energy) compared with quintile 1 (6.6–13.1% energy) was associated with higher self-reported physical function at baseline, slower rate of functional decline, higher GS * at baseline, slower declines in GS *, and more chair stands at baseline
Farsijani et al., 2016 [49]	Healthy community-dwelling men and women 67–84 y (712)	Protein quantity and distribution at meals at baseline and 2-year follow-up association with body composition	Men and women with evenly distributed protein intakes and men with high protein intakes showed higher LM or aLM throughout the entire follow-up period
Geirsdottir et al., 2013 [50]	Healthy community-dwelling men and women 65–92 y (237)	The association between dietary protein intake and body composition was measured	Mean protein intake was 0.98 ± 0.28 and 0.95 ± 0.29 g/kg body weight in male and female participants, respectively Dietary protein intake higher than RDA, was positively associated with LM
Granic et al., 2018 [51]	Community-dwelling men and women ≥ 85 y (722)	Evaluated associations between low protein intake (<1 g/kg aBW *) and changes in GS * and TUG *	Low protein intake associated with 1.62 kg lower baseline GS *, especially women, but rate of decline over 5 y not affected by protein status Women with low protein intake had worse baseline TUG, but rate of decline in TUG not affected by protein status
Gregorio et al., 2014 [52]	Healthy women 60–90 y (387)	Cross-sectional analysis of body composition and physical performance tests compared for those with protein intake below vs. at or above the RDA for protein (0.8 g/kg/d)	High protein group had lower total, fat, and lean mass and fat-to-lean ratio vs. lower protein group Upper and lower extremity function was impaired in low protein vs. high protein group
Hengeveld et al., 2021 [53]	Community-dwelling healthy men and women 67–84 y (1098)	Outcome measures included GS *, KES *, and physical performance (TUG *) Protein intake assessed via nine 24-h food records collected over 3 y	Higher daily protein intake was associated with better KES * and physical performance at 3 years in both genders and there was less physical performance decline in women In men, more uneven protein distribution was associated with better TUG * at 3 years and less GS * decline In women, higher number of protein snacks was associated with better GS * and KES * at 3 years and less GS * decline
Houston et al., 2008 [54]	Community-dwelling healthy men and women 70–79 y (2066)	The association between dietary protein intake and body composition was measured for 3-year changes. Quintiles for protein intake in g/d (Q1: 56.9 ± 18.6, Q2: 53.6 ± 19.8, Q3: 59.2 ± 18.2, Q4: 67.1 ± 19.2, Q5: 91.0 ± 27.1	Participants in the highest quintile of protein intake lost ~40% less LM and aLM than did those in the lowest quintile of protein intake
Isanejad et al., 2016 [55]	Women 65.3 to 71.6 y (554)	Cross-sectional and prospective study that assessed body composition and physical function Protein intake was grouped into lower (≤0.80 g/kg BM */d), moderate (PROT-AGE study group recommendation of 0.81–1.19 g/kg BM */d) or higher (≥1.2 g/kg BM */d)	At baseline, the higher protein group had better performance in the GS/BM *, KES/BM *, one-leg stance, chair rise, squat, squat to the ground, and had faster walking speed for 10 m and higher short physical performance battery vs. those with moderate and lower protein intakes At 3 y follow up, higher protein intake was associated with less decline in GS/BM *, one leg stance, and tandem walk for 6 m
Layman et al., 2004 [56]	Women 45–56 y with BMI * > 26 kg/m^2^ (24)	10-wk, 1700 kcal weight loss diet with either a carbohydrate/protein ratio of 3.5 (68 g protein/d; CHO group) or 1.4 (125 g protein/d; PRO group) with body composition and blood lipids measured	The PRO group lost 7.53 ± 1.44 kg body mass, while the CHO group lost 6.96 ± 1.36 kg body mass Weight loss in the PRO group had a higher proportion of fat/lean (6.3 ± 1.2 g/g) vs. the CHO group (3.8 ± 0.9 g/g) (*p* < 0.05)
Li et al., 2019 [57]	Men and women 40–80 y (3213)	Cross-sectional analysis in which dietary protein intake and body composition were obtained. Quintiles of protein intake were established (Q1: ≤0.96; Q2: 0.97–1.16; Q3: 1.17–1.38; Q4: 1.39–1.67; Q5: ≥1.68 g/kg/d).	The SMI * increased stepwise across percentiles in the fully adjusted model for relative total protein intake, relative animal protein intake, and relative plant protein intake (*P_trend_* < 0.001 in all cases) The odds of an individual having LMM * steadily decreased with each increase in total protein intake above Q1.
McLean et al., 2016 [58]	Men and women 29–85 y (1746)	Relationship between dietary protein (total, animal, and plant) and GS * was determined over 6 y follow up	Greater protein intake was associated with less decrease in GS *; ranging from lowest to highest quartiles of total protein intake, change in GS * (% per y) were −0.27, −0.15, 0.07, and 0.52). The trends for GS maintenance/improvement with higher protein intake were stronger for ages 60 + y vs. <60 y.
Nabuco et al., 2018 [59]	Healthy women ≥ 60 y (70)	Women resistance trained 3 days per wk for 12 wk. Women were assigned to: (1) 35 g hydrolyzed whey protein before each training session and carbohydrate placebo after (*n* = 24); (2) Carbohydrate placebo before and 35 g hydrolyzed whey protein after each training session (*n* = 23); or (3) Carbohydrate placebo before and after training (*n* = 23)	Protein supplementation increased total protein intake to 1.38 to 1.49 g/kg/d and each supplementation regimen equally increased energy intake from 22–23 kcal/kg to 26–28 kcal/kg Supplement timing relative to exercise did not affect the results, but whey protein hydrolysate supplementation improved SMM *, LLLST *, CP *, KES *, TS* and 10-m walk time vs. placebo only group
Oikawa et al., 2018 [60]	Healthy Men and women 68–69 y (31)	4-phase protocol: EB (1 wk): energy balance; 0.8 g/kg/d protein ER (1 wk): −500 kcal/d energy restriction; 1.6 g/kg/d protein (60 g/d whey or collagen peptides) ER + SR (2 wk): ER plus step reduction to <750/d RC (1 wk): Recovery of normal activity plus 1.6 g/kg/d protein (60 g/d whey or collagen peptides)	Higher protein intake did not protect against loss of leg lean mass from energy restriction or step reduction During RC, whey but not collagen: -Increased leg lean mass from ER + SR -Restored integrated muscle protein synthesis that had declined in ER and ER + SR
Park et al., 2018 [61]	Frail men and women 70–85 y (99)	In a 12-wk study, three protein intake groups: (1) 0.8 g/kg/d; maltodextrin powder; (2) 1.2 g/kg/d; combination of maltodextrin and whey protein powder; (3) 1.5 g/kg/d; combination of maltodextrin and whey protein powder	The 1.5 g/kg protein group, compared with the 0.8 g/kg protein group, had higher ASM *, ASM */weight, ASM */BMI *, ASM */fat ratio, and SMI *. Compared with the 0.8 g/kg protein group, the 1.5 g/kg protein group had improved gait speed.
Sahni et al., 2015 [62]	Healthy men and women 29–86 y (2675)	Protein intake, leg lean mass and isometric quadriceps strength were measured. Protein intake in g/d was split into quartiles for men and women, respectively—Q1: 64.9, 57.8; Q2: 70.8, 63.1; Q3: 79.2, 73.5; Q4: 101.1, 93.4	In both men and women, leg lean mass was higher in participants in the highest quartiles of total protein intake compared with those in the lowest quartiles of protein intake
Stookey et al., 2005 [63]	Healthy men and women 50–69 y (608)	Regression models used to determine if 3-day mean protein (% of energy) predicted changes in MAMA	Higher protein intake was associated with less loss of MAMA for both sexes
Vellas et al., 1997 [64]	Healthy men and women > 60 y (304)	Subjects were recruited into a 10-y longitudinal study to assess the relationships between nutrition and morbidity and mortality.	Women with protein intakes greater than the midrange of 0.8–1.2 g/kg body weight (1.20–1.76 g/kg) tended to have fewer health problems than those with protein intakes <0.8 g/kg

* Abbreviations: aBW = Adjusted Body Weight (for ideal weight); aLM = nonbone appendicular lean mass; ASM = Appendicular Skeletal Muscle Mass; BM = Body mass; BMI = Body Mass Index; CP = Chest Press Strength; CRP = C-reactive Protein; GS = Handgrip Strength; HDL = High Density Lipoprotein Cholesterol; IL-6 = Interleukin-6; KES = Knee Extension Strength; LLLST = Lower Limb Lean Soft Tissue; LM = Lean Mass; LMM = Low Muscle Mass; MAMA = Mid Arm Muscle Area; SMI = Skeletal Muscle Index; SMM = Skeletal Muscle Mass; TNF-α = Tumor Necrosis Factor-Alpha; TS = Total Strength (sum of chest press, preacher curl, knee extension); TUG = Timed Up and Go; WHI = Women’s Health Initiative.

**Table 3:** Comparison of Adult and Children RDA for Individual Amino Acids and Proposed Population Safe Estimates from IAAO Studies.

Amino Acid	Current Population Safe Intake for Adults (e.g., RDA or RNI, mg/kg BW/d) [3]	Current Population Safe Intake for Children (Boys & Girls 4–13 Years) (e.g., RDA or RNI, mg/kg BW/d) [3]	IAAO Proposed Population Safe Intake for Adults (e.g., RDA or RNI, mg/kg BW/d)	IAAO Proposed Population Safe Intake for Healthy Children 6–10 y (e.g., RDA or RNI, mg/kg BW/d)
Tryptophan, mg	5	6	5.0 [150]	6.1 [151]
Total Aromatic Amino Acids (TAA), mg	33	41	44–52 [146]	28 [145]
Total Sulfur Amino Acids (SAA), mg	19	22	21 [147]	17.9 [144]
Total Branched-chain Amino Acids (BCAA), mg	85	99	210 [148]	192 [143]
Lysine, mg	38	46	52.5 [152] 58.2 [27]	58 [141] 46.6 [142]
